# Pre-Synaptic Release Deficits in a DYT1 Dystonia Mouse Model

**DOI:** 10.1371/journal.pone.0072491

**Published:** 2013-08-13

**Authors:** Fumiaki Yokoi, Chad C. Cheetham, Susan L. Campbell, J. David Sweatt, Yuqing Li

**Affiliations:** 1 Department of Neurology, College of Medicine, University of Florida, Gainesville, Florida, United States of America; 2 Department of Neurology, Center for Neurodegeneration and Experimental Therapeutics, School of Medicine, University of Alabama at Birmingham, Birmingham, United States of America; 3 Department of Neurobiology, School of Medicine, University of Alabama at Birmingham, Birmingham, United States of America; University of Chicago, United States of America

## Abstract

DYT1 early-onset generalized torsion dystonia (DYT1 dystonia) is an inherited movement disorder caused by mutations in one allele of *DYT1* (*TOR1A*), coding for torsinA. The most common mutation is a trinucleotide deletion (ΔGAG), which causes a deletion of a glutamic acid residue (ΔE) in the C-terminal region of torsinA. Although recent studies using cultured cells suggest that torsinA contributes to protein processing in the secretory pathway, endocytosis, and the stability of synaptic proteins, the nature of how this mutation affects synaptic transmission remains unclear. We previously reported that theta-burst-induced long-term potentiation (LTP) in the CA1 region of the hippocampal slice is not altered in *Dyt1* ΔGAG heterozygous knock-in (KI) mice. Here, we examined short-term synaptic plasticity and synaptic transmission in the hippocampal slices. Field recordings in the hippocampal Schaffer collaterals (SC) pathway revealed significantly enhanced paired pulse ratios (PPRs) in *Dyt1* ΔGAG heterozygous KI mice, suggesting an impaired synaptic vesicle release. Whole-cell recordings from the CA1 neurons showed that *Dyt1* ΔGAG heterozygous KI mice exhibited normal miniature excitatory post-synaptic currents (mEPSC), suggesting that action-potential independent spontaneous pre-synaptic release was normal. On the other hand, there was a significant decrease in the frequency, but not amplitude or kinetics, of spontaneous excitatory post-synaptic currents (sEPSC) in *Dyt1* ΔGAG heterozygous KI mice, suggesting that the action-potential dependent pre-synaptic release was impaired. Moreover, hippocampal torsinA was significantly reduced in *Dyt1* ΔGAG heterozygous KI mice. Although the hippocampal slice model may not represent the neurons directly associated with dystonic symptoms, impaired release of neurotransmitters caused by partial dysfunction of torsinA in other brain regions may contribute to the pathophysiology of DYT1 dystonia.

## Introduction

Dystonia is a movement disorder characterized by sustained muscle contractions that often involve both agonist and antagonist muscles, causing twisting and repetitive movements or abnormal postures [1], [2]. Dystonia is caused by many etiologies; genetically, sporadically, or by other diseases, such as Parkinson’s disease, Huntington’s disease, brain injury, stroke, or drug side effects. Genetic dystonia is classified into more than 20 types, but only about half have been linked to a specific gene [3]–[6]. DYT1 early-onset generalized torsion dystonia [Oppenheim’s dystonia; Online Mendelian Inheritance in Man (OMIM) identifier #128100, Dystonia 1] is a genetic dystonia caused by mutations in *DYT1* (*TOR1A*) coding for torsinA with about 30% penetrance [7]. Symptoms of DYT1 dystonia usually appear in childhood or adolescence, first affecting limbs, and can become generalized [8]. The most common mutation is a trinucleotide deletion (ΔGAG), which causes a deletion of a glutamic acid residue (ΔE) in the C-terminal region [7]. In addition to the common ΔGAG mutation, an 18 bp-deletion mutation [9]–[11], an Arg288Gln missense mutation [12] and a frame-shift mutation caused by a 4 bp-deletion [13] have been reported in other dystonia families. Homozygous *DYT1* mutation carriers have not been reported in humans.

TorsinA belongs to the AAA^+^ (ATPases Associated with a variety of cellular Activities family of proteins) ATPase family, which is a large family of molecular chaperones [7]. Molecular chaperone-like activities of torsinA have been reported using *in vitro* or *in vivo* protein aggregation models. Overexpression of torsinA prevents aggregations of luciferase *in vitro* [14], accumulations of α-synuclein in cultured mammalian cells [15], and polyglutamine-repeat proteins in *Caenorhabditis elegans* [16], suggesting torsinA has molecular chaperone activities. TorsinA contributes to the stability of snapin, which functions in exocytosis [17]. Furthermore, torsinA binds to CSN4 and snapin in neuroblastoma cells and brain synaptosomes and may function in the synaptic protein stabilization and synaptic vesicle recycling [18]. The expression of genes associated with glutamate receptor-mediated synaptic plasticity is altered in cultured cell lines overexpressing human mutant torsinA [19], [20]. These studies suggest contribution of torsinA in protein processing through the secretory pathway [21], endocytosis [17], and stability of synaptic proteins [18]. Recently, an enhanced synaptic vesicle recycling [22] and more frequent miniature glutamate release [23] were reported in cultured neurons derived from the hippocampus of another line of ΔE-torsinA mouse. However, it is still not clear whether a similar alteration occurs in the brain circuits *in vivo*.

DYT1 dystonia is a neuronal circuit disorder rather than a neurodegenerative disorder [3]. Characterization of neuronal activities in the brain circuits is important to elucidate the mechanism of this disease. Electrophysiological recording in hippocampal slices is one of the established experimental models to examine long-term and short-term synaptic plasticity in the brain. Hippocampal CA3 pyramidal cells project to CA1 pyramidal cells through Schaffer collaterals (SC). We previously reported that theta-burst-induced LTP in the hippocampal CA1 region is not altered in *Dyt1* ΔGAG heterozygous KI mice [24]. Here, short-term synaptic plasticity of the same pathway was further examined in acute hippocampal slices. To characterize the stimulus-intensity dependent basal synaptic transmissions, input-output curves were obtained to measure the post-synaptic potential slope versus varying stimulus intensities. PPRs were further examined by field recordings of the SC pathway in various inter-stimulus intervals to analyze the probability of synaptic vesicle release and short-term plasticity. PPRs are inversely proportional to the probability of synaptic vesicle release [25]. When two consecutive action potentials are elicited in the presynaptic neuron, the amplitude of the second EPSC is inversely related to the amplitude of the first. Paired-pulse facilitation is observed when the first EPSC is smaller, *i.e*. the second EPSC is larger than the first, whereas paired-pulse depression is observed when the first EPSC is larger. PPRs at the SC synapse have been measured in hippocampal slices from various transgenic mouse lines to examine the probability of synaptic vesicle release [26]–[28].

Since these field recordings were derived by artificially evoked neuronal activities, spontaneous EPSCs (sEPSCs) of CA1 neurons were further recorded by whole-cell voltage-clamp under a GABA_A_ receptor blocker to analyze the spontaneous synaptic vesicle release. By blocking GABA_A_ receptors, activities of inhibitory neurons are suppressed, and spontaneous activities of CA1 neurons can be measured. Since sEPSCs are induced by both action-potential dependent and independent spontaneous releases, only action-potential independent spontaneous EPSCs, which are called miniature EPSCs (mEPSCs), were further analyzed by blocking voltage-dependent sodium channels in addition to GABA_A_ receptor blockade. These analyses elucidated a novel presynaptic deficit of hippocampal glutamate release in the DYT1 dystonia mouse model.

## Results

### No significant difference in input-output curves

**Figure 1:**
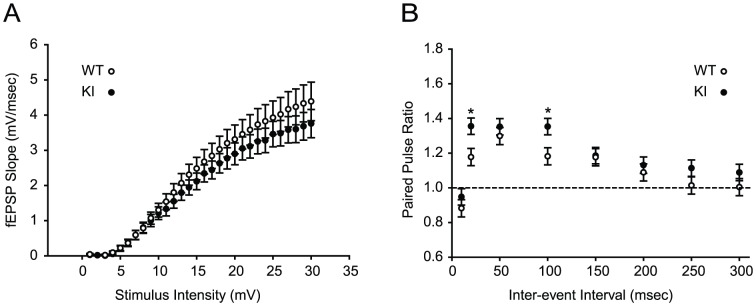
Enhanced PPRs in *Dyt1* ΔGAG heterozygous KI mice. (A) The input-output curves in *Dyt1* ΔGAG heterozygous KI mice were unaffected. (B) *Dyt1* ΔGAG heterozygous KI mice showed significantly enhanced PPRs at two inter-stimulus intervals (20 and 100 msec). Circles represent means ± standard errors. Open circles denote data from WT. Filled circles were from *Dyt1* ΔGAG heterozygous KI mice. *p<0.05.

We previously reported normal theta-burst-induced LTP of the CA1 region in the hippocampal slices from *Dyt1* ΔGAG heterozygous KI mice [24], suggesting long-term synaptic plasticity is not altered in this pathway. To characterize whether stimulus-intensity dependent basal synaptic transmission is altered, input-output curves were obtained by measuring the post-synaptic potential slope in varying stimulus intensities (Fig. 1A). *Dyt1* ΔGAG heterozygous KI mice showed no significant change in their input-output relationship from wild-type (WT) littermates (*p* = 0.39), suggesting that there is no significant difference in overall baseline synaptic transmissions.

### Enhanced PPRs in hippocampal slices from *Dyt1* ΔGAG heterozygous KI mice

Short-term plasticity was further evaluated by measuring PPRs at various inter-stimulus intervals. PPRs at two different inter-stimulus intervals were significantly enhanced in *Dyt1* ΔGAG heterozygous KI mice compared to WT littermates [Fig. 1B; means ± standard errors; 20 msec, WT, 1.177±0.050; KI, 1.355±0.047; *p* = 0.013; 100 msec, WT, 1.182±0.050; KI, 1.353±0.047; *p* = 0.016]. PPRs at other individual inter-stimulus intervals were higher on average in *Dyt1* ΔGAG heterozygous KI mice, but did not reach statistical significance. When all of the data was analyzed together, regardless of the inter-stimulus intervals, there was a trend of enhanced PPRs in *Dyt1* ΔGAG heterozygous KI mice compared to WT littermates (means ± standard errors; WT, 1.103±0.034; KI, 1.191±0.031; *p* = 0.089). Since PPRs are inversely proportional to the probability of synaptic vesicle release, the results suggest an impaired synaptic vesicle release in *Dyt1* ΔGAG heterozygous KI mice. However, PPRs were obtained by extracellular field recording of artificially evoked neurons, and it will be useful to explore this finding further in isolated synaptic pathways with intracellular recording configuration, which is much more sensitive and specific. Therefore, spontaneous neuronal activities were further analyzed using whole-cell patch clamp recording.

### Decreased frequency of sEPSCs in *Dyt1* ΔGAG heterozygous KI mice

**Figure 2:**
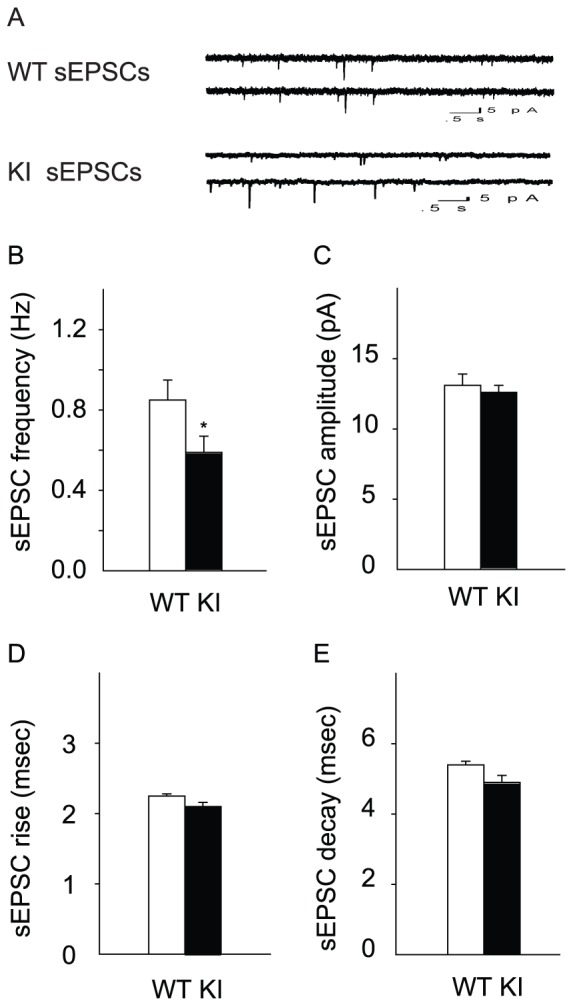
Decreased frequency of sEPSCs in *Dyt1* ΔGAG heterozygous KI mice. (A) Representative traces for sEPSCs. *Dyt1* ΔGAG heterozygous KI mice had a significantly decreased frequency of sEPSCs (B), but no change in either the amplitude (C), or rise (D) and decay (E) times of these events. The vertical bars represent means ± standard errors. *p<0.05.

Spontaneous excitatory post-synaptic currents (sEPSCs) from the CA1 pyramidal cells were obtained by a whole-cell voltage-clamp under a GABA_A_ receptor blocker to further explore the enhanced PPRs in *Dyt1* ΔGAG heterozygous KI mice (Fig. 2A). Both pre-synaptic and post-synaptic measurements were analyzed for sEPSCs. For pre-synaptic measurement, the frequency of the occurrence of sEPSCs was measured. The frequency of sEPSCs was significantly reduced in *Dyt1* ΔGAG heterozygous KI mice compared to that in WT littermates (Fig. 2B; WT, 0.85±0.10; KI, 0.59±0.08; *p*<0.05), suggesting pre-synaptic release was specifically reduced in *Dyt1* ΔGAG heterozygous KI mice. The post-synaptic measurements of amplitude and rise and decay times of these events were also measured. However, there was no significant change in sEPSC amplitude (Fig. 2C; WT, 13.1±0.8; KI, 12.6±0.5; *p*>0.05). In addition, no significant change in sEPSC rise (Fig. 2D; WT, 2.25±0.03; KI, 2.1±0.06; *p*>0.05) and decay (Fig. 2E; WT, 5.4±0.1; KI 4.9±0.2; *p*>0.05) times were detected between *Dyt1* ΔGAG heterozygous KI mice and WT littermates.

### Normal mEPSCs in *Dyt1* ΔGAG heterozygous KI mice

**Figure 3:**
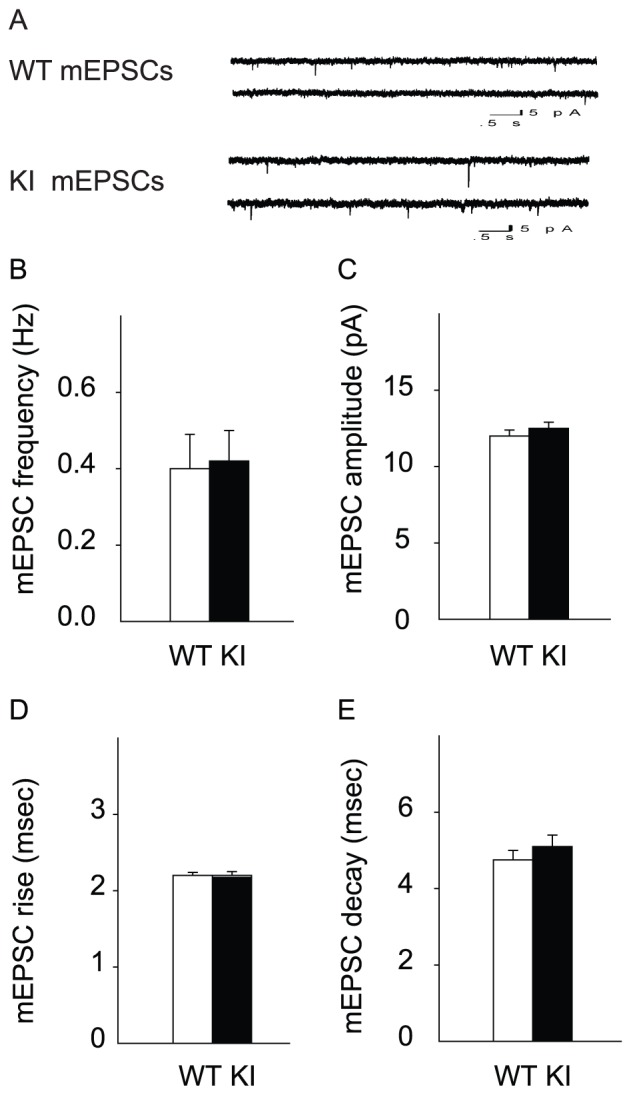
Normal mEPSCs in *Dyt1* ΔGAG heterozygous KI mice. (A) Representative traces for mEPSCs. *Dyt1* ΔGAG heterozygous KI mice had no change in frequency (B), amplitude (C), or rise (D) and decay (E) times of mEPSCs. The vertical bars represent means ± standard errors.

Since sEPSCs are the mixture of signals derived from action-potential dependent and independent spontaneous releases, only action-potential independent spontaneous EPSCs were further analyzed by blocking voltage-dependent sodium channels in addition to GABA_A_ receptors in order to determine which spontaneous release deficit causes the reduced frequency of sEPSCs. Both pre-synaptic and post-synaptic measurements were analyzed for miniature excitatory post-synaptic currents (mEPSCs; Fig. 3A). For pre-synaptic measurement, the frequency of the occurrence of mEPSCs was measured. In contrast to sEPSCs, there was no significant change in the mEPSC frequency in *Dyt1* ΔGAG heterozygous KI mice compared to WT littermates (Fig. 3B; WT, 0.40±0.09; KI, 0.42±0.08; *p*>0.05). The post-synaptic measurements of amplitude and rise and decay times of these events were also measured. Similar to sEPSCs, mEPSC amplitude (Fig. 3C; WT, 12.0±0.4; KI, 12.5±0.4; *p*>0.05), and rise (Fig. 3D; WT, 2.2±0.04; KI, 2.2±0.05; *p*>0.05) and decay (Fig. 3E; WT, 4.75±0.25; KI, 5.1±0.3; *p*>0.05) times were unchanged in *Dyt1* ΔGAG heterozygous KI mice. The mEPSCs data suggest that the action-potential independent spontaneous pre-synaptic release was normal. Taken together with the sEPSCs data, the results suggest that the action-potential dependent pre-synaptic release was specifically reduced in *Dyt1* ΔGAG heterozygous KI mice.

### Decreased hippocampal torsinA in *Dyt1* ΔGAG heterozygous KI mice

**Figure 4:**
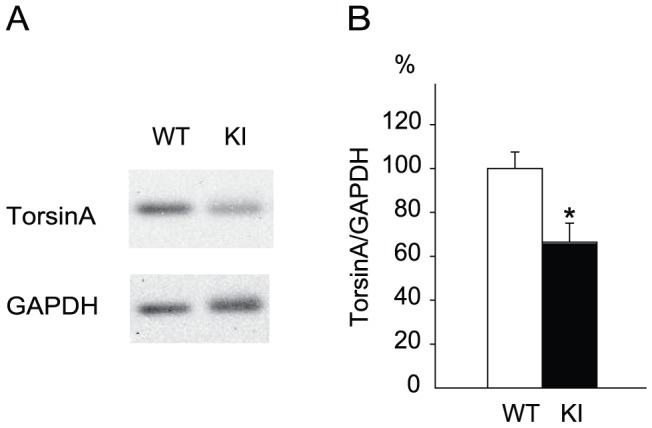
Reduced hippocampal torsinA in *Dyt1* ΔGAG heterozygous KI mice. A representative band image of western blot (A) and the quantified hippocampal torsinA levels (B) from WT and *Dyt1* ΔGAG heterozygous KI mice. The density of the torsinA band was standardized to that of GAPDH. The data in WT littermates were normalized to 100%. The vertical bars represent means ± standard errors. *p<0.05.

Reduced torsinA levels have been reported for whole brain [29], [30], striatum [31], liver [29] and embryonic fibroblast of *Dyt1* ΔGAG heterozygous KI mice [29]. However, the level of hippocampal torsinA has not been quantified in *Dyt1* ΔGAG heterozygous KI mice. To analyze the mechanism underlying the pre-synaptic release deficits caused by *Dyt1* ΔGAG mutation, we examined the hippocampal torsinA level. The hippocampi were dissected from *Dyt1* ΔGAG heterozygous KI mice and WT littermates, and their torsinA levels were examined by Western blot analysis (Fig. 4A). The torsinA level was significantly reduced in *Dyt1* ΔGAG heterozygous KI mice when compared to that in WT littermates (Fig. 4B; WT, 100±7.5%, n = 3; KI, 66.4±8.6%, n = 3; *p*<0.05). The results suggest that *Dyt1* ΔGAG heterozygous KI mice exhibit partial loss of torsinA function in the hippocampus.

## Discussion

Field recordings in the hippocampal SC pathway revealed significantly enhanced PPRs in *Dyt1* ΔGAG heterozygous KI mice, suggesting an impaired synaptic vesicle release. Whole-cell recordings from CA1 neurons revealed that *Dyt1* ΔGAG heterozygous KI mice exhibited normal mEPSCs, suggesting that the action-potential independent spontaneous pre-synaptic release was normal. On the other hand, there was a significant decrease in frequency, but not in amplitude or in kinetics, of sEPSCs in *Dyt1* ΔGAG heterozygous KI mice. Since the frequency of spontaneous transmitter release is determined pre-synaptically and amplitude and kinetics are determined post-synaptically, the results suggest that the action-potential dependent pre-synaptic release was impaired. The decreased frequency of sEPSCs may be caused by reduced action potential frequency in the pre-synaptic neurons, defective pre-synaptic response to the action potential, or both. Since *Dyt1* ΔGAG heterozygous KI mice showed a normal input-output relationship, the pre-synaptic functional alteration in *Dyt1* ΔGAG heterozygous KI mice is likely a property of the CA3 neurons or the group of neurons that control CA3 neurons’ excitability, either directly or indirectly. Spontaneous and evoked neurotransmitter releases are differentially regulated at central synapses [32]. The impaired pre-synaptic release we observed here is consistent with the reported association of torsinA with CSN4 and snapin, which is involved in synaptic vesicle release [17], [18], [33]. Moreover, hippocampal torsinA was significantly reduced in *Dyt1* ΔGAG heterozygous KI mice, suggesting that the impaired release of neurotransmitters may be caused by partial dysfunction of torsinA in the hippocampus.

LTP and PPR represent synaptic plasticity with different time spans. LTP refers to synaptic changes that last at least 30 minutes while PPR usually lasts less than 5 minutes. The mechanism of LTP and PPR are quite different and involve different molecular and cellular pathways [34]. The *Dyt1* KI mice showed normal LTP and enhanced PPR. This is similar to Cbl-b null mice [27], which exhibit enhanced PPRs in the SC glutamatergic synapses, while there is no alteration of LTP. On the other hand, Synaptotagmin IV null mice exhibit enhanced PPRs and increased LTP in hippocampus CA1, suggesting synaptotagmin IV, an activity-inducible secretory vesicle protein, regulates both LTP and short-term plasticity such as PPR [26]. Additionally, APPPS1-21 mice, an Alzheimer’s disease model, exhibit enhanced PPRs and reduced LTP [28]. These reports also suggest that the impaired short term plasticity, such as enhanced PPRs, does not necessarily associate with changes in LTP.

A previous study suggested more frequent miniature glutamate release in cultured neurons derived from the hippocampus of another line of KI mice [23]. However, mEPSCs were normal in our study using the brain slices. The reason for this discrepancy is not known, but may be due to differences in types of neurons recorded, cultured neurons versus brain slices, or the specific lines of KI mice used.

Since the hippocampus is a brain region that stores short-term spatial memory, pre-synaptic release deficits in the hippocampus itself should not contribute directly to dystonic symptoms. However, impaired release of neurotransmitters caused by partial dysfunction of torsinA in other brain regions may contribute to the pathophysiology of DYT1 dystonia. A transgenic mouse model, ectopically overexpressing human torsinA^ΔE^ derived by human CMV immediate early promoter, exhibits an imbalance between striatal dopaminergic and cholinergic signaling [35]. The transgenic mice also show a reduction of dopamine receptor 2 in the striatum and impaired LTD, and the LTD was rescued by an adenosine A2A receptor antagonist [36]. However, PPR was normal when measured in the corticostriatal pathway only at 50 msec inter-stimulus interval [35]. The corticostriatal LTD is also impaired in our *Dyt1* ΔGAG heterozygous KI male mice, and both LTD and motor deficits can be restored by trihexyphenidyl, an anticholinergic that is commonly used for DYT1 dystonia patients to release their dystonic symptoms [37]. Corticostriatal LTD is thought to involve a decrease of presynaptic glutamate release through retrograde endocannabinoid signaling [38]. Future studies focusing on the neurotransmitter release in the corticostriatal pathway will elucidate the contribution of neurotransmitter release in the pathophysiology of DYT1 dystonia.

TorsinA levels are reduced in DYT1 dystonia patient fibroblasts [29]. Consistent with humans, heterozygous *Dyt1* ΔGAG mutation causes a reduction of torsinA level in mouse brains [29], [30]. In adult *Dyt1* ΔGAG heterozygous KI mice, the striatal torsinA level is significantly reduced to 59.4%±8.4% [31]. Here, the hippocampal torsinA level was also significantly reduced in adult *Dyt1* ΔGAG heterozygous KI mice to 66.4±8.6% of that in WT littermates, suggesting that the heterozygous *Dyt1* ΔGAG mutation causes a similar reduction of torsinA, regardless of the brain region, in adult mice. TorsinA is stable and is degraded primarily through the macroautophagy-lysosome pathway, while torsinA^ΔE^ is quickly degraded via both the proteasome and macroautophagy-lysosome pathways, thus possibly explaining the reduced levels of torsinA in *Dyt1* ΔGAG heterozygous KI mice [39], [40]. Several lines of genetic mouse models also suggest that partial loss of torsinA function contribute to pathogenesis of DYT1 dystonia. For example, *Dyt1* knockdown male mice exhibit motor deficits similar to *Dyt1* ΔGAG heterozygous KI male mice [41]. Both cerebral cortex-specific and the striatum-specific *Dyt1* conditional knockout mice exhibit motor deficits similar to *Dyt1* ΔGAG heterozygous KI male mice [42], [43]. A chemical enhancement of torsinA levels restores the motor deficits in *Dyt1* ΔGAG heterozygous KI male mice [44]. Moreover, both *Dyt1* ΔGAG heterozygous KI mice and Purkinje cell-specific *Dyt1* conditional knockout mice exhibit similar alterations of dendritic morphology in the cerebellar Purkinje cells [45]. Moreover, ΔE-torsinA may affect WT torsinA function by inducing the disassembly of WT torsinA oligomers as shown by an *in vitro* experiment [46]. Future studies using *Dyt1* heterozygous KO mice will elucidate the effect of partial loss of torsinA function on the action-potential dependent pre-synaptic release.

AAA^+^ ATPases are present in both pre- and post-synaptic neurons and contribute to short-term or long-term synaptic plasticity. *N*-ethylmaleimide-sensitive factor (NSF) is a member of AAA^+^ ATPase family and is involved in synaptic vesicle fusion and recycling in cooperation with α-SNAP, SNAREs and other proteins. It also contributes to calcium-dependent neurotransmitter release from presynaptic neurons [47]. NSF binds to AMPA receptor GluR2, disassembles the GluR2-PICK1 complex in postsynaptic neurons, and regulates long-term synaptic plasticity [48]. Similarly, AAA^+^ ATPase Thorase mediates the internalization of AMPA receptors by disassembling the AMPA receptor-GRIP1 complex and regulates AMPA receptor-dependent long-term synaptic plasticity [49]. The present results suggest that torsinA, a member of AAA^+^ ATPase family, may directly regulate action potential-dependent neurotransmitter release similar to NSF. Moreover, partial loss of torsinA may affect the neurotransmitter release through the interaction with proteins, such as CSN4 and snapin [17], [18], [33], that are known to play important roles in vesicle release. Finally, since the majority of torsinA is supposed to be localized in the endoplasmic reticulum and nuclear envelope, torsinA may indirectly influence neurotransmitter release through its involvement in maturation and trafficking of synaptic proteins [21], [50]. Identification of the specific cellular pathways that are altered in presynaptic neurons in mutant mice will elucidate the molecular mechanism of torsinA in regulating neurotransmitter release.

## Materials and Methods

### Mice

All experiments were carried out in compliance with the U.S. Public Health Service Commissioned Corps (USPHS) Guide for Care and Use of Laboratory Animals and approved by the Institutional Animal Care and Use Committee (IACUC) at the University of Alabama at Birmingham (UAB) with Animal Protocol Number 091008198. *Dyt1* ΔGAG heterozygous KI mice were prepared and genotyped by PCR as previously described [24], [51]. For the following experiments, only male mice were used. All mice were housed under a 12-hour light, 12-hour dark cycle with *ad libitum* access to food and water.

### Preparation of hippocampal slices

Field recordings were performed in 6 *Dyt1* ΔGAG heterozygous KI mice and 5 WT littermates as described earlier [52]. Hippocampi of adult KI or WT mice were rapidly removed and briefly chilled in ice-cold cutting saline (110 mM sucrose, 60 mM NaCl, 3 mM KCl, 1.25 mM NaH_2_PO_4_, 28 mM NaHCO_3_, 5 mM D-glucose, 500 µM CaCl_2_, 7 mM MgCl_2_, and 600 µM ascorbate). Transverse 400-µm thick slices were prepared with a vibratome and maintained at least 45 min in a holding chamber containing 50% artificial cerebral spinal fluid (aCSF; 125 mM NaCl, 2.5 mM KCl, 1.25 mM NaH_2_PO_4_, 25 mM NaHCO_3_, 25 mM D-glucose, 2 mM CaCl_2_, and 1 mM MgCl_2_) and 50% cutting saline. The slices were then transferred to a recording chamber and perfused (1 ml/min) with 100% aCSF. Slices were allowed to equilibrate for 60–90 min in a Fine Science Tools interface chamber at 30°C. All solutions were continuously bubbled with 95% O_2_/5% CO_2_.

### Set-up and electrode placement for field recordings

For extracellular field recordings, glass recording electrodes were pulled from capillary glass tubes using a horizontal electrode puller (Narishige) and filled with aCSF. The input resistance of each electrode was tested by applying a current pulse and breaking the tip until a resistance of 1–3 MΩ was obtained. Recording electrodes were placed in the stratum radiatum of hippocampal area CA1. Test stimuli were delivered to the Schaffer collateral/commissural pathway with bipolar Teflon coated platinum stimulating electrode positioned in stratum radiatum of area CA3. Responses were recorded through a personal computer using AxoClamp pClamp8 data acquisition software. Excitatory Post-Synaptic Potential (EPSP) slope measurements were taken after the fiber volley to eliminate contamination by population spikes.

### Input-output curves

Test stimuli were delivered and responses were recorded at 0.05 Hz; every six consecutive responses over a 2 min period were pooled and averaged. fEPSPs were recorded in response to increasing intensities of stimulation (from 2.5 µA to 45.0 µA). Averages at individual stimulus intensities were compared.

### Paired pulse ratios

PPRs were measured at various inter-stimulus intervals (10, 20, 50, 100, 150, 200, 250, and 300 msec). All experimental stimuli were set to an intensity that evoked 50% of the maximum fEPSP slope. Averages were compared using n (number of slices) as the level of analysis.

### Whole-cell recordings

A Zeiss Axioskop FS microscope (Zeiss, Thornwood, NY, USA), equipped with Nomarski optics, 40× water immersion lens and infrared illumination were used to view neurons in the slices. aCSF was heated to 32–35°C by an in-line heater (Warner Instruments, Hamden, CT). The temperature was monitored by a thermometer placed in the recording chamber. Cells were labeled intracellularly with biocytin (0.5%; Sigma, St. Louis, MO, USA) and processed as previously described [53] to confirm identification. Whole-cell voltage-clamp recordings were obtained as described previously [53]. Patch electrodes had an open tip resistance of 3–4 MΩ. Tight seals (>2 GΩ before breaking into whole-cell mode) were obtained under visual guidance. Series resistances during recording were allowed to vary from 10 to 20 MΩ and were not compensated. Recordings were terminated whenever significant increases (>20%) in series resistance occurred. The intracellular solution for recordings contained 125 mM K gluconate,10 mM KCl, 10 mM HEPES, 2 mM Mg-ATP, 0.2 mM Na-GTP, and 0.5 mM EGTA. Osmolarity and pH were adjusted to 290 mOsm and 7.3, respectively. All recordings were made in the presence of 10 µM bicuculline (Sigma, St. Louis, MO) in the bath solution to block GABA_A_ receptors. For recording miniature Excitatory Post-Synaptic Currents (mEPSCs), 1 µM tetrodotoxin (TTX; Sigma, St. Louis, MO) was also added to the bath solution to block voltage-dependent sodium channels. Spontaneous and mEPSCs were analyzed using the Mini Analysis program from Synaptosoft, Inc. (Decatur, GA, USA). Frequency, amplitude, rise and decay times of sEPSCs and mEPSCs were averaged across animals of the same genotype and compared using the number of neurons (n) as the level of analysis.

### Western blot for torsinA

Protein extracts were prepared from 3 *Dyt1* ΔGAG heterozygous KI mice and 3 WT littermates as described earlier [31]. The hippocampi were dissected and quickly frozen in liquid nitrogen. The hippocampi were then homogenized in 400 µl of ice-cold lysis buffer [50 mM Tris·Cl (pH 7.4), 175 mM NaCl, 5 mM EDTA•2Na, complete Mini (Roche)] and sonicated for 10 sec. One-ninth volume of 10% Triton X-100 in lysis buffer was added to the homogenates. The homogenates were incubated for 30 min on ice and then centrifuged at 10,000×*g* for 15 min at 4°C. The supernatants were then collected, and the protein concentration was measured by Bradford assay with bovine serum albumin (Fisher Scientific) as standards [54]. The homogenates were mixed with sodium dodecyl sulfate polyacrylamide gel electrophoresis (SDS-PAGE) loading buffer, boiled for 5 min, incubated on ice for 1 min, and then centrifuged for 5 min to obtain the supernatant. Forty µg of total protein was separated by SDS-PAGE and transferred to PROTRAN nitrocellulose transfer membranes (Whatman). The membranes were blocked in 5% milk (Bio-rad) in TBS-T buffer [20 mM Tris·Cl (pH 7.6), 137 mM NaCl, 0.1% (v/v) Tween 20] for 1 hour at room temperature. The membranes were incubated with rabbit anti-torsinA antibody (Abcam; ab34540) in 5% milk TBS-T buffer overnight at 4°C. The membranes were washed in TBS-T buffer, incubated with bovine anti-rabbit IgG-HRP (Santa Cruz; sc-2370) in 5% milk TBS-T buffer at room temperature for 1 hour, and washed. TorsinA bands were detected by SuperSignal West Pico Chemiluminescent Substrate (Thermo Scientific), and the signal was captured by an Alpha Innotech FluorChem FC2 camera. To detect Glyceraldehyde-3-phosphate dehydrogenase (GAPDH) as a loading control, the torsinA antibody was stripped off from the membranes in Restore Western Blot Stripping buffer (Thermo Scientific). The membranes were washed in TBS-T buffer and re-blocked in 5% milk TBS-T buffer. The membranes were incubated with Horseradish peroxidase (HRP)-conjugated anti-GAPDH (Santa Cruz; sc-25778 HRP) at 4°C overnight. After washing with TBS-T buffer, GAPDH bands were detected as described above. The density of each band was quantified with UN-SCAN-IT gel (Silk Scientific) software. Molecular masses were estimated with Precision Plus Protein Standards All Blue (Bio-Rad). Western blot analysis was performed in triplicate. The density of the torsinA band was standardized to that of GAPDH. The data in WT littermates were normalized to 100%, and those in *Dyt1* ΔGAG heterozygous KI mice were compared.

### Statistics

The fEPSP slope data was analyzed by the Kolmogorov-Smirnov test in SAS/STAT Analyst software (Version 9.1.3; SAS Institute Inc. NC). Paired Pulse Ratios were analyzed by ANOVA mixed model in the software with repeated measurements for either data at each inter-event interval or all data, regardless of the inter-event intervals. The frequency, amplitude, and kinetics of sEPSC and mEPSC, as well as the hippocampal torsinA levels, were compared between *Dyt1* ΔGAG heterozygous KI mice and WT littermates using Student’s t-test. Significance was assigned at *p*<0.05.
